# Clinical outcome with different doses of low-molecular-weight heparin in patients hospitalized for COVID-19

**DOI:** 10.1007/s11239-021-02401-x

**Published:** 2021-03-01

**Authors:** Marco G. Mennuni, Giulia Renda, Leonardo Grisafi, Andrea Rognoni, Crizia Colombo, Veronica Lio, Melissa Foglietta, Ivan Petrilli, Mario Pirisi, Enrico Spinoni, Danila Azzolina, Eyal Hayden, Gianluca Aimaretti, Gian Carlo Avanzi, Mattia Bellan, Vincenzo Cantaluppi, Andrea Capponi, Luigi M. Castello, Damiano D’Ardes, Francesco Della Corte, Sabina Gallina, Marco Krengli, Mario Malerba, Sante D. Pierdomenico, Paola Savoia, Patrizia Zeppegno, Pier P. Sainaghi, Francesco Cipollone, Giuseppe Patti, the COVID-UPO Clinical Team

**Affiliations:** 1 Azienda Ospedaliero-Universitaria Maggiore della Carità, Novara, Italy; 2 G. d’Annunzio University, Chieti-Pescara, Italy; 3 Ospedale Santissima Annunziata of Chieti, Chieti, Italy; 4 Dipartimento di Medicina Traslazionale, Università del Piemonte Orientale, UPO, Azienda Ospedaliero-Universitaria Maggiore della Carità, Via Solaroli 17, 28100 Novara, Italy; 5 Sant’Andrea Hospital, Vercelli, Italy

**Keywords:** COVID-19, Enoxaparin doses, Mortality, Thromboprophylaxis

## Abstract

A pro-thrombotic *milieu* and a higher risk of thrombotic events were observed in patients with CoronaVirus disease-19 (COVID-19). Accordingly, recent data suggested a beneficial role of low molecular weight heparin (LMWH), but the optimal dosage of this treatment is unknown. We evaluated the association between prophylactic vs. intermediate-to-fully anticoagulant doses of enoxaparin and in-hospital adverse events in patients with COVID-19. We retrospectively included 436 consecutive patients admitted in three Italian hospitals. Outcome according to the use of prophylactic (4000 IU) vs. higher (> 4000 IU) daily dosage of enoxaparin was evaluated. The primary end-point was in-hospital death. Secondary outcome measures were in-hospital cardiovascular death, venous thromboembolism, new-onset acute respiratory distress syndrome (ARDS) and mechanical ventilation. A total of 287 patients (65.8%) were treated with the prophylactic enoxaparin regimen and 149 (34.2%) with a higher dosing regimen. The use of prophylactic enoxaparin dose was associated with a similar incidence of all-cause mortality (25.4% vs. 26.9% with the higher dose; OR at multivariable analysis, including the propensity score: 0.847, 95% CI 0.400–0.1.792; p = 0.664). In the prophylactic dose group, a significantly lower incidence of cardiovascular death (OR 0.165), venous thromboembolism (OR 0.067), new-onset ARDS (OR 0.454) and mechanical intubation (OR 0.150) was observed. In patients hospitalized for COVID-19, the use of a prophylactic dosage of enoxaparin appears to be associated with similar in-hospital overall mortality compared to higher doses. These findings require confirmation in a randomized, controlled study.

**Supplementary Information:**

The online version contains supplementary material available at 10.1007/s11239-021-02401-x.

## Highlights

- Thrombosis and inflammation have an interacting and circular relationship in COVID-19.
- In patients with COVID-19, a hypercoagulable state justifies the use of heparin as prophylaxis for future thrombotic events.
- A prophylactic dosage of enoxaparin seems to be associated with similar in-hospital mortality compared to higher doses

## Introduction

Coronavirus Disease-2019 (COVID-19), caused by Severe Acute Respiratory Syndrome CoronaVirus-2 (SARS-CoV-2) infection, was first described in China in December 2019; since then, it has rapidly become a global pandemic [1]. Patients hospitalized for COVID-19 usually present a respiratory syndrome, but several reports have described an increased occurrence of macrovascular thrombotic complications, with attendant higher mortality [2, 3]. Indeed, autoptic data also indicated a diffuse microvascular thrombosis in the lungs of patients who died for COVID-19 [4–6]. Thus, based on such evidence and on real-world data suggesting a clinical benefit of anticoagulant therapy [7–10], patients hospitalized for COVID-19 commonly receive early treatment with low molecular weight heparin (LMWH). Although the World Health Organization supported a strategy with LMWH at prophylactic dosage (4000 IU daily) [11], in patients admitted for COVID-19 it is common practice to use intermediate-to-therapeutic (i.e., fully anticoagulant) doses of LMWH. This is in agreement with recent reports showing a pro-thrombotic *milieu* in such patients [2, 12]. Thus, due to the lack of specific comparison data, the optimal dosage of LMWH in this setting is unknown and debated. In this retrospective, multicenter study, we specifically investigated the in-hospital outcome with prophylactic vs. higher dosage of LMWH in patients hospitalized for COVID-19.

## Methods

### Study population

Consecutive patients aged ≥ 18 years and admitted for COVID-19 from February 20 to May 12, 2020, in three Italian Hospitals (Maggiore della Carità Hospital of Novara; Sant’Andrea Hospital of Vercelli; Policlinico Santissima Annunziata of Chieti) were included. All patients had a nasopharyngeal swab tested positive for molecular detection of SARS-CoV-2 RNA by reverse-transcriptase–polymerase-chain-reaction assay.

**Fig. 1:**
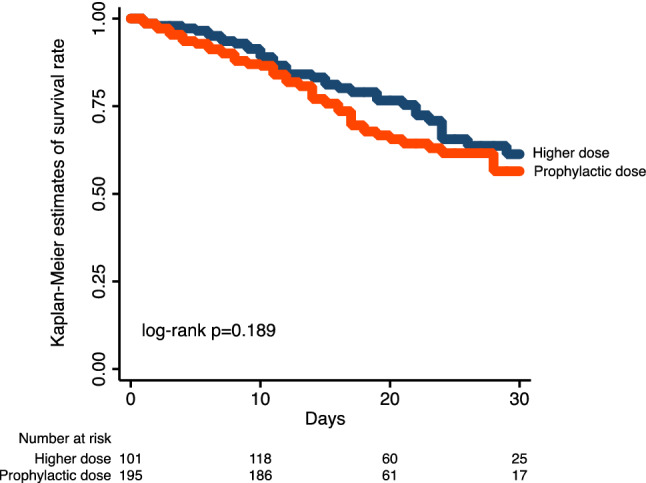
Kaplan–Meier estimates of 30-day survival stratified by different doses of enoxaparin

A total of 637 patients with COVID-19 were admitted during the study period. For the purpose of the study, we considered those patients receiving LMWH at any time during the in-hospital stay. Two-hundred-one patients were excluded from the analysis: 157 patients because they did not receive LMWH due to clinical decision of the treating physician; 23 patients due to lack of information on the LMWH dose; 4 patients who received thrombo-prophylaxis with fondaparinux; 17 patients treated with oral anticoagulant agents. Thus, a total of 436 patients represent the study population. The flow diagram indicating how the final number of included patients was obtained is reported in Fig. 1 of the Online Appendix. The choice of dosing regimen of LMWH and timing of LMWH initiation was left to the physician’s discretion; however, as per internal in-hospital protocols, patients deemed suitable for pharmacological prevention of thrombotic events generally received subcutaneous LMWH early after the admission, and LMWH was enoxaparin in all cases. No antiplatelet therapy or unfractionated heparin were given for thrombo-prophylaxis; unfractionated heparin was utilized only in patients with thrombotic complications during hospitalization. In the analysis, no patient had received both LMWH doses; switching from prophylactic to higher enoxaparin doses was performed only in the case of in-hospital thrombotic complication. Patients were included regardless of clinical features at presentation and in-hospital therapies for COVID-19. The Institutional Review Board approved the study protocol (IRB code CE 97/20), which was conducted in strict accordance with the Declaration of Helsinki principles.

### Data collection

We identified patients hospitalized for COVID-19 from hospital administrative data. An electronic case report form was generated using the Research Electronic Data Capture software (REDCap, Vanderbilt University), where individual data obtained after the revision of clinical records were entered. The data entry was retrospectively performed by investigators involved in the patient’s management. A unique pseudonymized code was assigned to each patient. Individual data included patients’ demographic details, comorbidities, vital signs, laboratory test results, medications and in-hospital events.

### Investigational regimens exposure and study end-points

Patients were divided according to the received enoxaparin dose: prophylactic (4000 IU daily) vs. higher dosage (> 4000 IU daily). The primary end-point was the incidence of all-cause death during in-hospital stay in the two groups (prophylactic vs. higher dosing regimen).

The following in-hospital secondary end-points were considered:

- Cardiovascular mortality, defined as death resulting from an acute myocardial infarction, heart failure, stroke, pulmonary embolism, or other cardiovascular causes [13].
- Venous thromboembolism, including pulmonary embolism or deep venous thrombosis
- New-onset severe acute respiratory distress syndrome (ARDS), defined according to the Berlin definition, as an acute (within a week), diffuse, inflammatory lung injury, not fully explained by cardiac failure or fluid overload, leading to increased pulmonary vascular permeability and loss of aerated lung tissue acute, with bilateral lung opacities consistent with pulmonary edema and ratio of the partial pressure of arterial oxygen to the fraction of inspired oxygen (PaO_2_/FiO_2_) < 100 mmHg [14].
- Need for mechanical ventilation. Criteria for mechanical ventilation were: cardiac or respiratory arrest; inability to protect the airway; coma or psychomotor agitation; unmanageable secretions or uncontrolled vomiting; life-threatening arrhythmias or electrocardiographic signs of ischemia; hemodynamic instability, defined as systolic arterial pressure less than 90 mmHg despite adequate filling or use of vasoactive agents; intolerance to all interfaces; dyspnea during noninvasive continuous positive airway pressure, a respiratory rate more than 30 breaths/min; peripheral oxygen saturation below 92% during noninvasive continuous positive airway pressure; acidosis with a pH < 7.35.
- Major bleeding and clinically relevant non-major bleeding, according to the International Society of Thrombosis and Haemostasis (ISTH) definition [15]. This analysis was performed on a subgroup of 86 consecutive patients whose bleeding complications were systematically collected and validated.

All outcome events (both primary and secondary) were counted if they occurred after the admission and after the initiation of LMWH treatment. Patients without adverse events who were still hospitalized at the time of the analysis were not included in the analysis. The length of stay was reported as days from admission to discharge.

### Statistical analysis

Continuous data are reported as mean ± standard deviation if normally distributed, or as median and interquartile range if not normally distributed, and were compared by Student t-test or Mann–Whitney U test, as appropriate. Categorical variables are indicated as number (percentage), and proportions were compared by chi-squared test. Frequencies were calculated to examine the associations between LMWH doses and outcome.

**Fig. 2:**
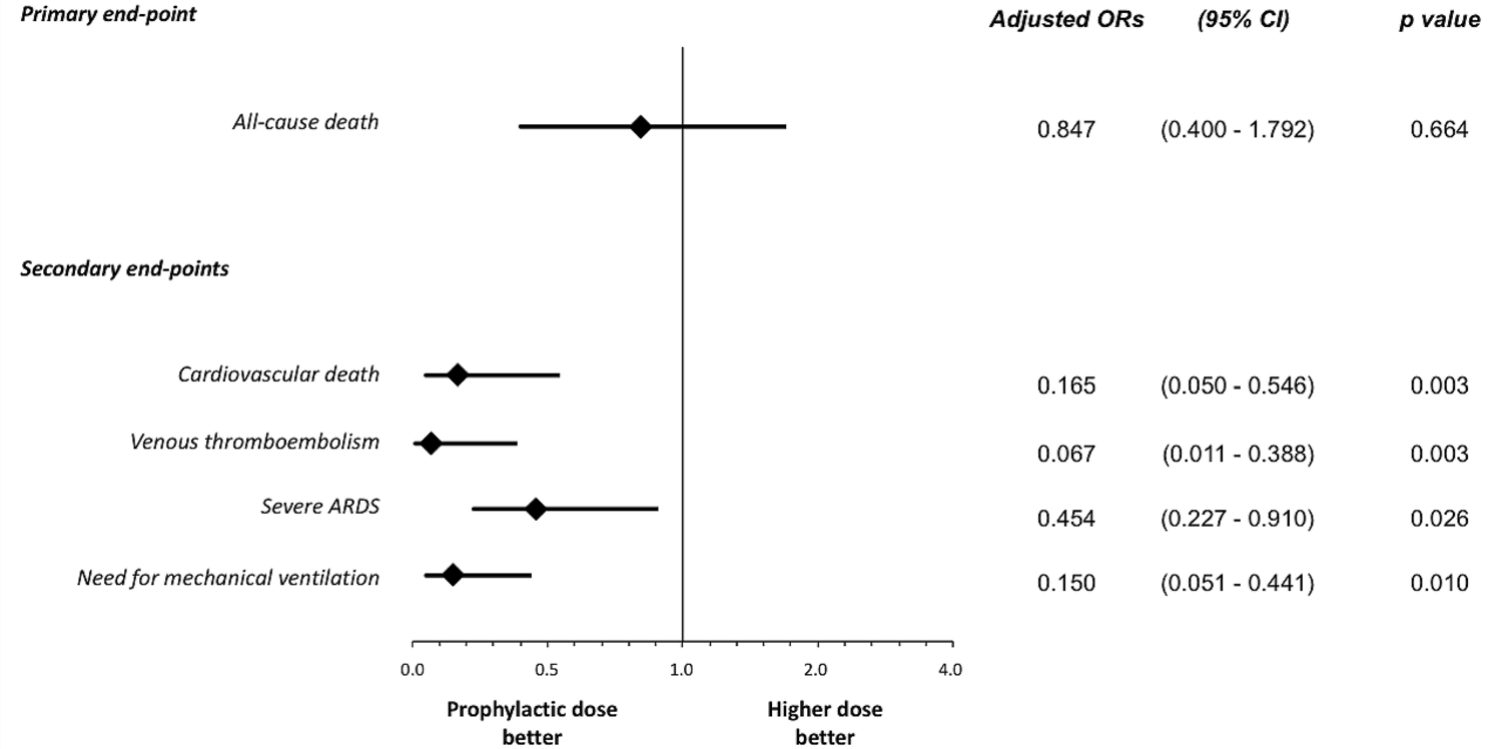
Adjusted ORs for the study end-points with prophylactic vs. higher enoxaparin dose. *ARDS* acute respiratory distress syndrome, *CI* confidence interval, *OR* Odds ratio

Logistic regression models were used to estimate the independent association between LMWH dosing regimens and study end-points. Odds ratios (OR) and 95% confidence interval (CI) were calculated. The residual imbalance of adjusting covariates was addressed by a “doubly robust” method, incorporating relevant covariates in two regression models: a propensity-score model and an outcome regression model [16]. The first model was fitted to account for the non-randomized use of different LMWH dosages. The individual propensities for receiving a prophylactic LMWH dose were estimated through a logistic regression model, including baseline demographic/clinical characteristics and comorbidities (see Tables 1 and 2). We then added the propensity score based on patients’ characteristics as an additional covariate into the outcome regression model (Fig. 2 of the Online Appendix). This latter model included demographic factors (age, gender, body weight), clinical factors (comorbidities, chronic use of antiplatelet or oral anticoagulant therapy), laboratory findings (PaO_2_/FiO_2_) and in-hospital medications for COVID-19. In order to adjust for timing of LMWH initiation, early administration of LMWH (< 24 h from admission) was also added as a covariate.

**Table 1:** Baseline characteristics of patients stratified by enoxaparin dose

	Prophylactic dose N = 287	Higher dose N = 149	p value
Demographic			
Age (years)	71.2 ± 15.6	70.2 ± 13.0	0.175
Male gender	159 (55.4)	90 (60.4)	0.525
Body weight (kg)	72.6 ± 13.3	79.3 ± 13.6	< 0.001
Comorbidities			
Arterial hypertension	152 (53.0)	93 (62.4)	0.059
Diabetes mellitus	66 (23.0)	29 (19.5)	0.397
Obesity^a^	34 (14.7)	33 (27.5)	0.004
Smoking	40 (13.9)	20 (13.4)	0.882
Heart failure	41 (14.3)	27 (18.1)	0.156
Atrial fibrillation	22 (7.7)	34 (22.8)	< 0.001
Peripheral vascular disease	45 (15.7)	20 (12.4)	0.530
Chronic obstructive pulmonary disease	35 (12.2)	12 (8.1)	0.186
Chronic renal failure	51 (17.8)	17 (11.4)	0.083
History of cancer	56 (19.5)	25 (16.8)	0.486
Chronic liver disease	10 (3.5)	3 (2.0)	0.392
Clinical signs upon presentation			
Systolic blood pressure (mmHg)	129.0 ± 22.2	130.2 ± 22.1	0.591
Heart rate (bpm)	83.7 ± 14.3	87.3 ± 21.0	0.060
Body temperature (°C)	37.5 ± 1.1	37.4 ± 1.1	0.425
Severe ARDS at ED presentation	12 (4.2)	16 (10.7)	0.008
Laboratory findings			
White blood cells (n/mm^3^)	7757 ± 5652	8460 ± 5245	0.113
Hemoglobin (g/dL)	12.8 ± 2.0	12.9 ± 2.1	0.601
Platelets (n/mm^3^)	213,140 ± 99,410	223,030 ± 97,938	0.383
C-reactive protein (mg/dL)	23.8 ± 43.6	22.4 ± 38.7	0.336
Creatinine (mg/dL)	1.3 ± 1.3	1.0 ± 0.6	0.044
d-Dimer (µg/L)^a^	4,233 ± 10,227	5,183 ± 13,902	0.616
PaO_2_/FiO_2_ ratio	284.5 ± 100.2	235.4 ± 93.1	< 0.001
Risk of death upon presentation			
4C Mortality Score			0.230
Low (0–3)	12 (4.2)	2 (1.3)	
Intermediate (4–8)	90 (31.4)	46 (30.9)	
High (9–14)	168 (58.5)	96 (64.4)	
Very high (> 14)	17 (5.9)	5 (3.4)	

Data are expressed as n (%) or mean ± standard deviation. *ARDS* acute respiratory distress syndrome; *ED* emergency department

^a^Data were missing for obesity in 85 patients (56 in the prophylaxis and 29 in the higher dose group) and D-dimer in 267 patients (196 in the prophylaxis and 71 in the higher dose group)

**Table 2:** Medical treatment in patients stratified by enoxaparin dose

	Prophylactic dose N = 287	Higher dose N = 149	p value
Medical therapy			
ACE-inhibitors	58 (27.2)	40 (31.0)	0.454
Sartans	47 (22.2)	35 (27.3)	0.280
Aspirin	60 (28.0)	40 (32.0)	0.440
P_2_Y_12_ inhibitors	24 (11.4)	17 (13.4)	0.583
Oral anticoagulants	15 (7.0)	32 (24.8)	< 0.001
COVID-19 treatment			
Hydroxychloroquine	185 (84.9)	117 (9.7)	0.118
Daunavir	75 (35.5)	48 (39.0)	0.525
Remdesivir	0	6 (4.9)	0.001
Lopinavir	33 (15.6)	29 (23.6)	0.069
Lopinavir/Ritonavir	31 (15.2)	29 (25.2)	0.028
Azitromicin	76 (35.3)	44 (36.4)	0.852
Steroids	80 (36.7)	70 (58.8)	< 0.001
Tocilizumab	19 (9.2)	25 (21.7)	0.002

Data are expressed as n (%)

*ACE* Angiotensin-converting enzyme, *COVID-19* CoronaVirus disease-19

A subgroup analysis was additionally performed to test the interaction between earlier administration of LMWH and mortality with different doses of LMWH. With the aim to keep into account differences in follow-up duration between the two study groups, a time-to-event analysis was performed for the primary end-point by using the time on LMWH treatment as a time-dependent variable. An estimation of survival at 30 days was calculated by the Kaplan–Meier method, with differences between different LMWH dosing regimens being assessed by log-rank test.

Two sensitivity analyses were conducted. We evaluated the primary outcome with different LMWH dosages stratifying by the risk of death at hospital presentation using the 4C Mortality Score; it classifies the risk of death of COVID-19 patients, based on clinical features on admission, into four groups: low, intermediate, high and very high risk [17]. Thus, we estimated the association of 4000 IU daily dose of enoxaparin vs. > 4000 IU daily dose for the primary end-point in the subgroups with low/intermediate and high/very high risk. Moreover, to test the relationship between thrombo-prophylaxis and outcome at the extreme of body weight (obesity or underweight), we performed a sensitivity analysis in the subgroup of patients in whom a precise body weight was identified on the source documents (N = 212). We classified the body weight-adjusted enoxaparin dose as prophylactic (≤ 70 IU/kg daily) or higher (> 70 IU/kg daily) and evaluated the association of the two regimens with the primary end-point. A logistic regression analysis was also performed in the two sensitivity estimations, as previously indicated for the primary assessments. Statistical analyses were performed by Stata software, version 16.0.

## Results

A total of 287 patients (65.8%) received a prophylactic enoxaparin dose (4000 IU daily) and 149 (34.2%) a higher enoxaparin dose (> 4000 IU daily). The distribution of baseline characteristics, clinical presentation, comorbidities, concomitant treatments and laboratory findings according to the enoxaparin dosing regimen is shown in Tables 1 and 2. A prophylactic enoxaparin dose was associated with lower body weight, reduced prevalence of atrial fibrillation and severe ARDS at Emergency Department presentation, as well as with higher creatinine values and increased PaO_2_/FiO_2_ at baseline_._ Treatment with remdesivir, lopinavir/ritonavir, steroids and tocilizumab was more frequent in patients receiving the higher enoxaparin dosage. The length of in-hospital stay was shorter in patients receiving the prophylactic dose (Table 3).

**Table 3:** In-hospital outcome according to different enoxaparin doses

	Prophylactic dose N = 287	Higher dose N = 149	p value
Primary end-point			
All-cause death	73 (25.4)	40 (26.9)	0.750
Secondary end-points			
Cardiovascular death	11 (3.9)	19 (12.8)	0.001
Venous thromboembolism	3 (1.1)	19 (12.8)	< 0.001
New-onset severe ARDS	56 (19.5)	74 (49.7)	< 0.001
Need of mechanical ventilation	15 (5.2)	38 (25.5)	< 0.001
Major bleeding^a^	1 (1.5)	1 (4.8)	0.431
Clinically relevant non-major bleeding^a^	2 (3.1)	1 (4.8)	0.999
In-hospital length of stay (days)^b^	13 (8–18)	17 (11–25)	< 0.001

*ARDS* acute respiratory distress syndrome

^a^Bleeding was assessed in a subgroup including 65 consecutive patients in the prophylaxis and 21 patients in the higher dose group

^b^Expressed as median (interquartile range)

A total of 113 patients (25.9%) had a primary end-point event. Crude rates of the study end-points are reported in Table 3. Patients on prophylactic enoxaparin dose had a comparable incidence of the primary outcome measure of all-cause mortality (25.4% vs. 26.9% in those receiving the higher dose; p = 0.750). The estimated survival rates at 30 days among patients on prophylactic vs higher dosing regimen were 56.5% (95% CI 45.7–65.9%) and 61.3% (95% CI 49.3–71.3%), respectively (log-rank *p* = 0.189; Fig. 1). Multivariate analysis, also adjusted for the propensity score, indicated a similar risk of in-hospital death in the two treatment groups (OR 0.847, 95% CI 0.400–1.792, p = 0.664) (Fig. 2). In the overall population, 68.9% of patients received enoxaparin < 24 h from Emergency Department presentation and 80.8% of patients < 48 h. There was no interaction between earlier initiation of enoxaparin (< 24 h from admission) and different doses of enoxaparin in terms of in-hospital mortality (earlier prophylactic dose 24.8%; earlier higher dose 26.3%; late prophylactic dose 27.8%; late higher dose 25.6%; p for interaction = 0.726).

All secondary outcome measures were associated with a significantly lower risk in the prophylactic dose group (Fig. 2); in particular, adjusted OR for cardiovascular mortality was 0.165 (95% CI 0.050–0.546, p = 0.003), adjusted OR for venous thromboembolism was 0.067 (95% CI 0.011–0.388, p = 0.003), adjusted OR for new-onset severe ARDS was 0.454 (95% CI 0.227–0.910, p = 0.026) and for mechanical ventilation was 0.150 (95% CI 0.051–0.441, p = 0.010). The incidence of bleeding events was low in both groups, without significant differences (major bleeding: 1.5% with the prophylaxis regimen vs. 4.8% with the higher dose regimen; clinically relevant non-major bleeding: 3.1% vs. 4.8%).

**Fig. 3:**
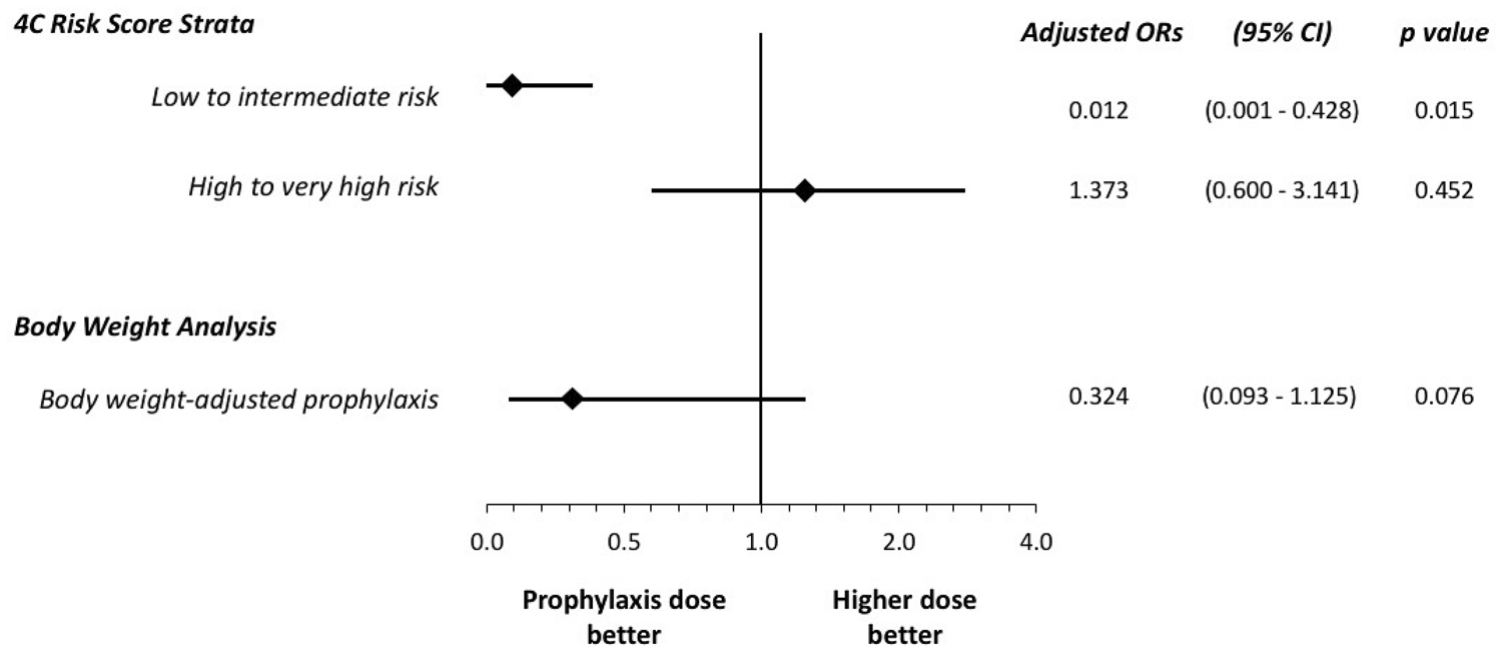
Adjusted ORs for the primary end-point with: 4000 IU vs. > 4000 IU enoxaparin daily dose in the subgroups with low/intermediate and high/very high risk according to the 4C Mortality Score; ≤ 70 IU/Kg vs. > 70 IU/Kg enoxaparin daily regimen. *CI* Confidence interval; *OR* Odds ratio

Sensitivity analysis showed that, as compared to the higher dosing regimen, the use of prophylactic dosage of enoxaparin was associated with comparable outcome for the primary end-point in patients (N = 286) with high/very high risk of death on admission (adjusted OR 1.373, 95% CI 0.600–3.141, p = 0.452) and an improved outcome in those (N = 150) with low/intermediate risk (adjusted OR 0.012, 95% CI 0.001–0.428, p = 0.015) (p for interaction 0.02) (Fig. 3). The analysis according to body weight-adjusted enoxaparin dose showed a similar incidence of the primary end-point in patients receiving ≤ 70 IU/Kg vs. > 70 IU/Kg daily regimen (adjusted OR 0.324, 95% CI 0.093–1.125, p = 0.076) (Fig. 3).

## Discussion

In this analysis, involving a multicenter cohort of patients admitted for COVID-19, the use of enoxaparin at prophylactic doses, as compared with higher doses, was associated with similar in-hospital mortality.

COVID-19 is a complex disease with both a primary involvement of the respiratory system and a pro-coagulative status [2, 3, 12]. As a matter of fact, autoptic studies on COVID-19 cadavers showed both micro-vascular lung thrombosis and macrovascular systemic or pulmonary thrombosis, regardless of antithrombotic treatment, type of anticoagulant therapy, and timing of disease course [4–6]; this suggests a pivotal role of coagulative patterns in the disease process. The indication from the World Health Organization on the use of prophylactic LMWH doses in patients with COVID-19 [11] was essentially based on preliminary evidence showing a benefit compared to no thrombo-prophylaxis, mainly in subgroups at higher risk [8, 9]; such recommendation was consistent with pre-existing guidelines for thrombo-prophylaxis in high-risk acutely ill patients [18–20]. However, small observational studies demonstrated rates > 20% of thrombotic complications among critically ill patients with COVID-19, despite standard thrombo-prophylaxis [3, 9, 21]. Although flawed by the use of heterogeneous agents and variable dosages, these reports generated a source of concern and encouraged the use of intermediate or full-dose anticoagulation in this setting. Two recent investigations from the same study group showed a survival benefit of full-dose anticoagulation in patients hospitalized for COVID-19 [7], especially when initiated > 48 h from admission [22]; these results are challenging to interpret and export in clinical practice, given the mixing of oral, subcutaneous or intravenous agents and the absence of specific information on treatment indication and dosages. Thus, given the lack of direct comparisons between prophylactic and higher regimens, the optimal dosage of LMWH for preventing thrombotic events in patients with COVID-19 is uncertain and is a matter of active debate.

We explored this issue in a consecutive cohort of patients hospitalized in three Italian Hospitals, in whom a specific comparison of two dosages of enoxaparin (4000 IU vs. > 4000 IU daily) was performed. Notably, regardless of the dosing strategy, in our series enoxaparin was early given (< 48 h from admission in 81% of patients). Patients receiving the higher enoxaparin dosing regimen had higher body weight, increased frequency of atrial fibrillation, more severe hypoxemia upon admission, and received a “more aggressive” treatment for COVID-19; the length of stay was also longer. On the other hand, patients treated with the prophylaxis regimen had a higher prevalence of risk factors increasing the COVID-19-related mortality, i.e. older age, diabetes mellitus, chronic obstructive pulmonary disease, renal failure and cancer [23]. Thus, to reduce residual confounding and limit the bias from unbalanced baseline features in the study population, we performed a logistic regression analysis, where the propensity score was also added as covariate. Here the use of prophylactic and higher dose of enoxaparin resulted in a similar incidence of in-hospital mortality, even after adjustment for the timing of drug initiation. The consistency of results in the sensitivity analysis among higher risk patients strengthens the robustness of the primary analysis by further minimizing the risk of treatment bias, at least in this more critical setting. These findings lead support to the recent decision of the United States National Institutes of Health to stop the ongoing randomized trial on anticoagulants in high-risk patients with COVID-19 for futility and potential harm [24]. Notably, in our study, the use of prophylactic doses of enoxaparin in patients at low or intermediate baseline risk was associated with reduced in-hospital death, but this merits further confirmation in a larger, specific investigation.

The concomitant reduction of new-onset severe ARDS and venous thromboembolism in patients treated with prophylaxis regimen supports that both lung microvascular angiopathy and macrovascular venous thrombosis have a predominant role in the pathogenesis and natural history of COVID-19 [2–6]. Prevention of these thrombotic phenomena may reduce the need for mechanical ventilation and translate into a significant benefit in terms of lower cardiovascular death, as observed in the present study. According to our results, in the context of patients with COVID-19 (i.e., characterized by mounting inflammation, cytokine storm and imbalance of the hemostatic system), an approach of full anticoagulation with therapeutic doses of LMWH could not be optimal. Notably, COVID-19-related lung microvascular angiopathy shares similar pathogenetic mechanisms with complement‐mediated thrombotic microangiopathy [5, 25, 26] and here full dose anticoagulation yielded unsatisfactory results [25]. Various features may explain the reason of why, in the absence of indication to therapeutic dosages for an active macrovascular thrombosis, dosing regimens of LMWH higher than prophylactic in patients with COVID-19 are associated with a poorer outcome: increased risk of heparin-induced thrombocytopenia, leading to further enhancement of the pro-thrombotic status; lower “pleiotropic” (anti-inflammatory and immunomodulant) effects [27]; increased risk of intraparenchymal bleeding and hemorrhagic infarctions in the lung, already described in COVID-19 pathology findings and possibly underestimated/undiagnosed in the COVID-19 scenario [28]. However, these hypotheses require specific evaluations in ad hoc*,* mechanistic studies.

We performed an additional sensitivity analysis by comparing prophylactic vs. higher body weight-adjusted enoxaparin doses (≤ 70 IU/Kg vs. > 70 IU/Kg daily). It confirmed no advantage of higher enoxaparin dosages in terms of all-cause death. Thus, our data support a standard approach of 4000 IU enoxaparin daily dose, with a strategy of body weight-adjusted dosing regimen (70 IU/Kg daily) being used in patients at the extremes of body weight. This approach is in agreement with the recommendation of the European Society of Cardiology expert consensus on LMWH dosing [29], suggesting that fixed prophylactic doses should be utilized in patients with normal weight, overweight and mild obesity; LMWH dose should be adjusted for the body weight only in patients with underweight or severe obesity. Indeed, in patients with COVID-19, a body weight-adjusted dosing appears crucial mainly in obese patients, where an additional mortality risk and a higher intrinsic risk of thromboembolic complications have been described [30, 31].

The present study confirms previous reports indicating a low incidence of hemorrhagic complications in patients with COVID-19 and reinforces the concept that the thrombotic risk rather than bleeding risk is the major concern in this setting [7]. In our investigation, the low rates of both major bleeding and clinically relevant non-major bleeding preclude any definite conclusion on the risk of hemorrhagic events with prophylactic vs. higher enoxaparin doses.

### Study limitations

The study has strengths and limitations. A multicenter cohort of consecutive patients was included, and individual data were accurately collected, with a strict source verification for the event adjudication. However, the retrospective design and the conduction of the study during a National Emergency contributed to the lack of some, although limited, data, which were not available. Given the observational nature, the study should not be used to definitely rule out either benefit or harm of different enoxaparin dosing regimens. However, the consistency of the results across multiple adjustments and analyses is reassuring for the robustness of our findings. Nevertheless, the risk of unmeasured confounding remains. Finally, our results specifically refer to enoxaparin and whether they are also generalizable to other LMWH molecules is unknown.

In conclusion, a prophylactic dose of enoxaparin in patients admitted for COVID-19 is associated with similar in-hospital mortality compared to higher dosing regimens. Our findings are hypothesis-generating and require confirmation in a randomized, controlled study.

## Supplementary Information

Below is the link to the electronic supplementary material.

Supplementary file1 (PPTX 37 KB)
Flow diagram showing how the final study population was obtained. LMWH= Low molecular weight heparin

Supplementary file2 (PPTX 67 KB)
Propensity score histogram by different dosing regimens of enoxaparin
