# Supplementary material for: Clinical outcome with different doses of low-molecular-weight heparin in patients hospitalized for COVID-19
Source: J Thromb Thrombolysis. 2021 Mar 1;52(3):782–90. doi: 10.1007/s11239-021-02401-x (PMC7919624; doi:10.1007/s11239-021-02401-x)
Supplement: Supplementary file 1 — Supplementary file1 (PPTX 37 KB) Flow diagram showing how the final study population was obtained. LMWH= Low molecular weight heparin [file 11239_2021_2401_MOESM1_ESM.pptx]

## Slide 1
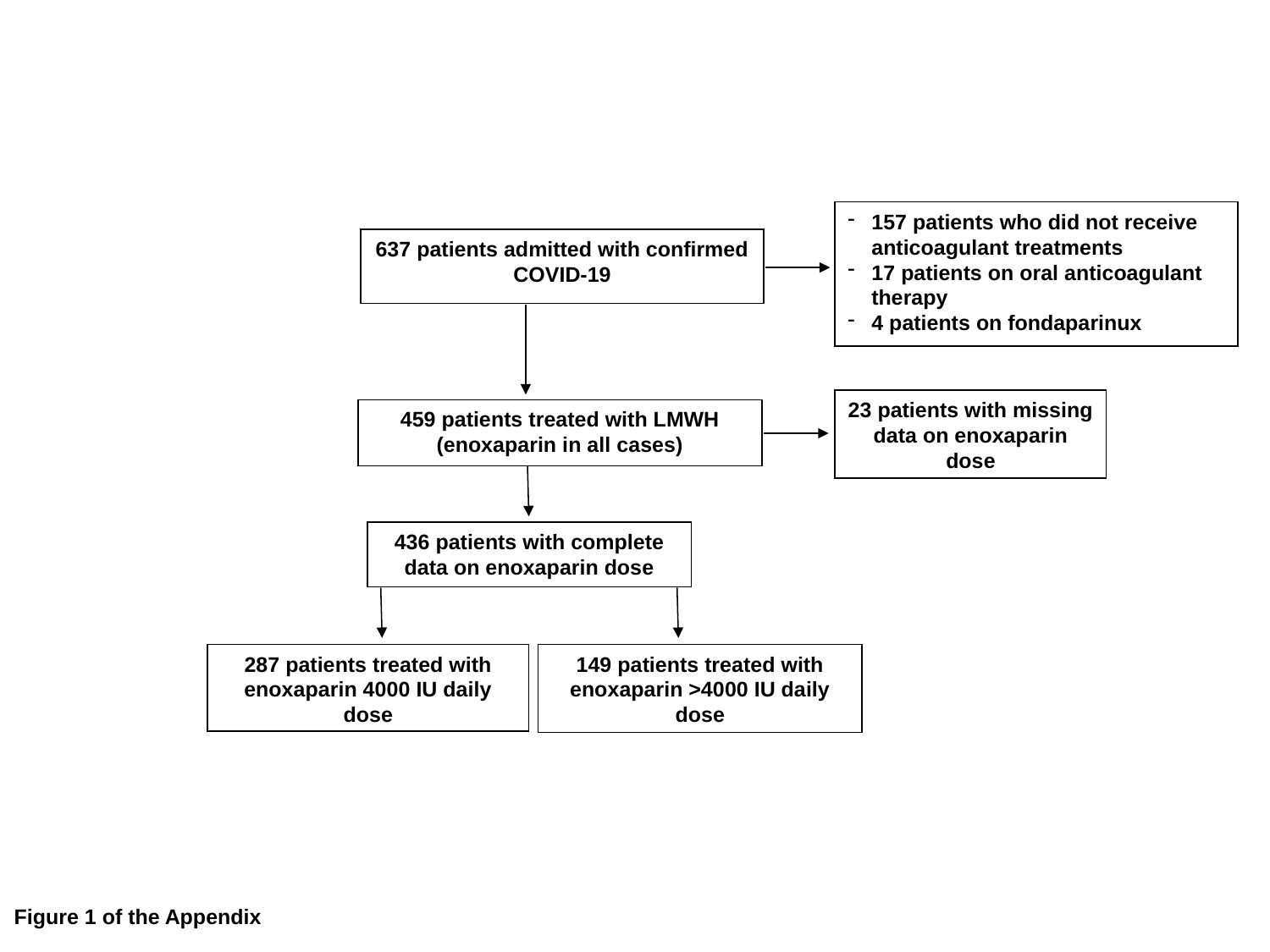

157 patients who did not receive anticoagulant treatments
17 patients on oral anticoagulant therapy
4 patients on fondaparinux
637 patients admitted with confirmed COVID-19
23 patients with missing data on enoxaparin dose
459 patients treated with LMWH (enoxaparin in all cases)
436 patients with complete data on enoxaparin dose
149 patients treated with enoxaparin >4000 IU daily dose
287 patients treated with enoxaparin 4000 IU daily dose
Figure 1 of the Appendix
