# Supplementary figures and images for: Clinical outcome with different doses of low-molecular-weight heparin in patients hospitalized for COVID-19
Source: J Thromb Thrombolysis. 2021 Mar 1;52(3):782–90. doi: 10.1007/s11239-021-02401-x (PMC7919624; doi:10.1007/s11239-021-02401-x)

## Slide 1
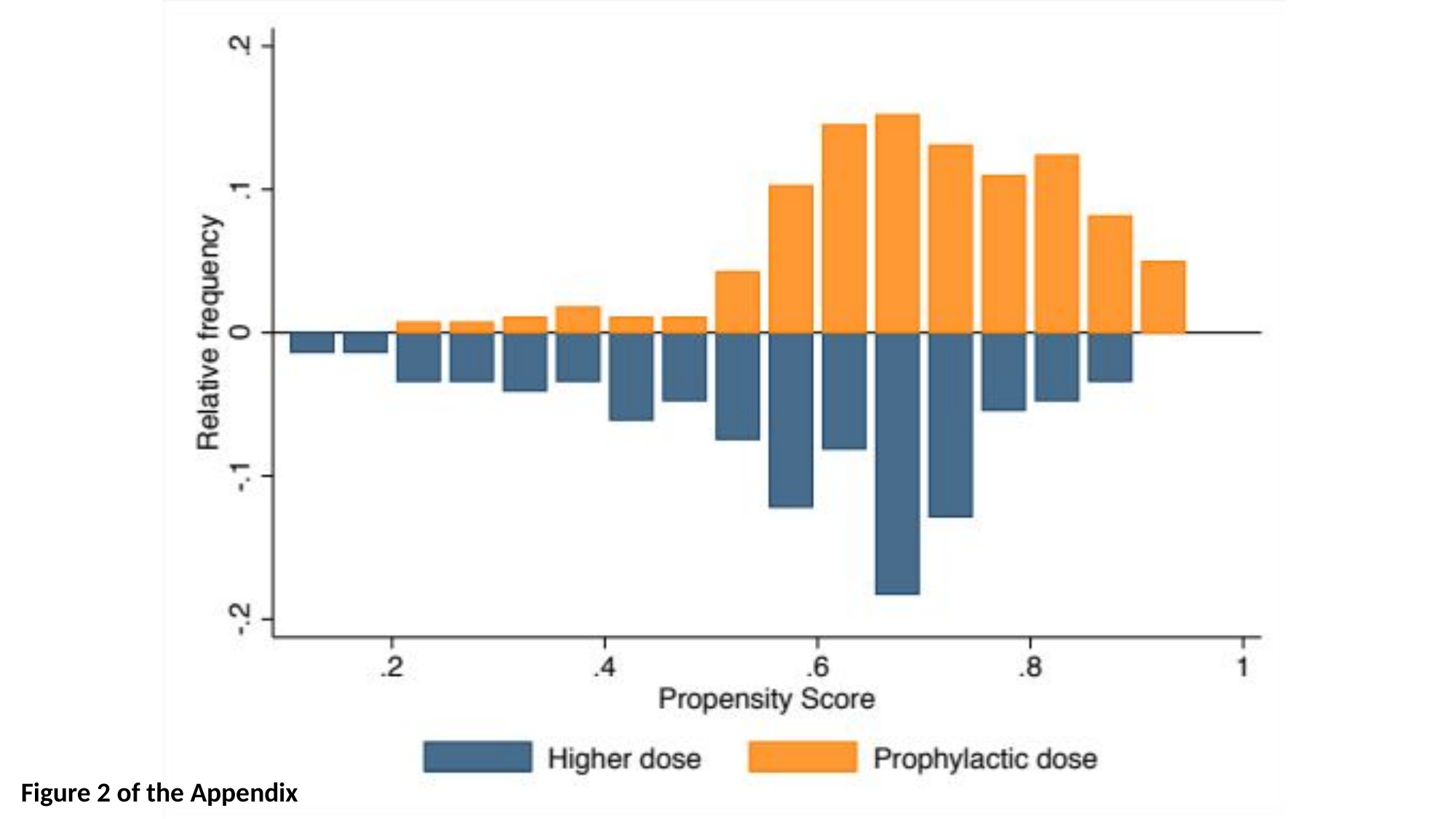

Figure 2 of the Appendix

Supplement: Supplementary file 2 — Supplementary file2 (PPTX 67 KB) Propensity score histogram by different dosing regimens of enoxaparin [file 11239_2021_2401_MOESM2_ESM.pptx]
